# Bacterial extracellular vesicles: emerging mediators of gut-liver axis crosstalk in hepatic diseases

**DOI:** 10.3389/fcimb.2025.1620829

**Published:** 2025-06-20

**Authors:** Yutong Zhou, Yong Sun, Pengsheng Yin, Shi Zuo, Haiyang Li, Kun Cao

**Affiliations:** ^1^ Department of Hepatobiliary Surgery, The Affiliated Hospital of Guizhou Medical University, Guiyang, Guizhou, China; ^2^ Key Laboratory of Hepatobiliary and Pancreatic Diseases Treatment and Bioinformatics Research, Guizhou Medical University, Guiyang, China; ^3^ Guizhou Medical University, The Provincial Key Laboratory of Digestive System Diseases of Guizhou Province, Guiyang, China

**Keywords:** bacterial extracellular vesicles, gut-liver axis, liver diseases, MAFLD/MASH, liver fibrosis, HCC

## Abstract

Bacterial Extracellular Vesicles (BEVs) are key mediators of cross-talk between gut microorganisms and host organs, playing an especially important role in the gut-liver axis. In this paper, we systematically review the mechanisms of BEV production, their classification, and their regulatory networks in liver diseases. BEVs carry pathogenic factors such as lipopolysaccharide (LPS) and bacterial DNA, which can enter the circulatory system by disrupting the intestinal barrier and target the liver to induce metabolic abnormalities, including insulin resistance. Furthermore, through activation of signaling pathways such as LPS/TLR4, cGAS/STING, and TGF-β, BEVs promote the progression of metabolism-associated fatty liver disease (MAFLD), liver fibrosis, and hepatocellular carcinoma. In addition, BEVs show dual potential in the diagnosis and treatment of liver diseases: on one hand, they can be used as non-invasive biomarkers to enhance diagnostic specificity through multi-omics analysis; on the other hand, engineered and modified BEVs, as well as probiotic BEVs (e.g., from *Lactobacillus* and *Bifidobacterium* species), can regulate lipid metabolism, reduce inflammation, and even enhance immunotherapy by targeting the tumor microenvironment. However, the heterogeneity of BEVs, efficient isolation techniques, storage stability, and clinical translation remain major challenges in current research. In the future, combining multi-omics techniques to resolve the molecular fingerprints of BEVs, optimizing isolation methods, and exploring their potential as precision medicine tools will be necessary to advance the study of the gut-liver axis toward clinical applications.

## 1 Background

As the largest metabolic organ in the body, the liver collects blood from the portal venous system, including nutrients, microbial metabolites, and potential pathogens (Gilgenkrantz, et al., 2025). Existing studies have suggested that the gut interacts with the liver through the gut-liver axis and that metabolites of the gut microbiota can influence liver homeostasis, thereby modulating metabolic function, immune status, and inflammatory responses (Hsu and Schnabl, 2023). In a healthy state, the host prevents microorganisms and their metabolites from entering the circulation through the intestinal barrier and the liver’s defense mechanisms (Gilgenkrantz, et al., 2025). However, in liver disease, an imbalance of intestinal flora and damage to the intestinal barrier can lead to the translocation of pathogenic factors, such as bacterial metabolites and bioactive substances, into the bloodstream, which can further exacerbate liver injury (Tilg et al., 2022). Currently, therapeutic strategies for liver disease mainly include removal of the cause, pharmacological interventions (e.g., antifibrotic, antioxidant, and immunomodulatory therapies), and surgical treatments, such as liver transplantation for end-stage patients (Zhang et al., 2023). Although these measures have achieved some success in slowing disease progression and improving survival, they still face many challenges in promoting overall disease resolution. Existing treatments mostly focus on etiologic control or management of advanced complications, and there is a lack of targeted interventions that can effectively block or reverse chronic inflammation, immune imbalance, and fibrotic processes (Gilgenkrantz et al., 2025). In addition, the pathogenesis of liver diseases is highly complex, involving multiple components such as metabolic disorders, inflammatory responses, impaired immune regulation, and abnormalities in the gut-liver axis, which further limits the overall benefit of current therapeutic approaches (Hasa et al., 2022). Therefore, developing novel intervention strategies targeting the core pathological aspects of inflammation, immunity, and fibrosis is a key direction for urgent breakthroughs in the future treatment of liver diseases. In recent years, breakthroughs in technologies such as microbial metagenome sequencing and metabolite assays, in addition to transcriptome, proteome, and metabolome analyses, complemented by pathology studies using animal models, have dramatically improved our understanding of the composition of the microbiome and the pathogenesis of liver disease.

The complex and dynamic interaction mechanisms between the human microbiota and the host primarily involve metabolic pathways and signaling networks mediated by bioactive molecules. Some bioactive molecules are packaged into nanoparticles called extracellular vesicles (EVs). EVs are believed to be spontaneously secreted membranous vesicular structures of all cells, including eukaryotic and prokaryotic cells, that carry a wide range of biomolecules (proteins, lipids, nucleic acids, etc.) and are widely involved in intercellular communication, tissue repair, immunoregulation, and other biological processes (Buzas, 2023). Recently, it has been found that extracellular vesicles secreted by bacteria play an important role in host-microbe interactions and disease regulation (Toyofuku et al., 2019; Tiku and Tan, 2021). Bacterial extracellular vesicles (BEVs) are nanometer-sized membranous particles with diameters ranging from 20 to 400 nm, produced by Gram-negative or Gram-positive bacteria. Similar to eukaryotic EVs, BEVs are capable of carrying a variety of biomolecules from the organism itself as well as from the living environment and act as “messengers” involved in inter-bacterial communication and cross-border regulation between bacteria and host cells (Toyofuku et al., 2019; Amalia and Tsai, 2023; Wang et al., 2023). In recent years, there has been a growing interest in the complex relationship between the microbiota and liver disease. Most studies have focused on the hepatic effects of diet-dependent metabolites produced by the gut microbiota (e.g., short-chain fatty acids, tryptophan metabolites, trimethylamine-N-oxides, bile acids, etc.). In contrast, fewer studies have explored the underlying mechanisms by which BEVs exert their effects, i.e., the microbial metabolites themselves. However, the available data confirm that the half-life of bacterial-associated metabolites is usually short and that host immune mechanisms also degrade biomolecules produced by bacteria, such as proteins and nucleic acids, so it cannot be fully assumed that interactions between microorganisms and other organs rely solely on the secretion of these bacterial bioactives without the need for protective transport via carriers (Rooks and Garrett, 2016; Lavelle and Sokol, 2020). Thus, microbial effects on the liver are at least partially mediated by their BEVs. In this review, we will summarize BEVs’ origin, circulation and distribution pathways and highlight their multifaceted roles in pathogenesis as well as in the diagnosis and management of liver diseases. Finally, we will discuss the current limitations of BEVs research and suggest potential future research directions.

## 2 Overview of bacterial extracellular vesicles

### 2.1 Mechanism of production and classification of bacterial extracellular vesicles

Gram-negative and Gram-positive bacteria differ in their biological origins and the substances they carry due to differences in their surface structure. Gram-negative bacteria produce extracellular vesicles by membrane vesiculation and cell blast lysis. Bacterial outer membrane vesicles (OMVs) derived from outer membrane vesicles consist of outer membrane and periplasmic components such as LPS. Similarly, outer-inner membrane vesicles (OIMVs) are formed by an autolysin-mediated blistering mechanism with a weakened peptidoglycan layer and contain the outer and inner membranes of the mother cell as well as cytoplasmic components. Degradation of the bacterial peptidoglycan layer by phage-derived endolysins leads to explosive cell lysis and random encapsulation of cytoplasmic contents in explosive outer membrane vesicles (EOMVs) and explosive outer-inner membrane vesicles (EOIMVs). These subtypes are often collectively referred to as OMVs because of the difficulty in accurately differentiating them. Gram-positive bacteria produce cytoplasmic membrane vesicles (CMVs) that lack an outer membrane because of the absence of an outer membrane structure. Thus, more cytoplasmic membrane vesicles are produced, and the production mechanism is similar to that observed in Gram-negative bacteria as part of explosive cell death. They can be categorized into cytoplasmic membrane vesicles (CMVs), explosive cytoplasmic membrane vesicles (ECMVs), and CMVs produced by a vesicular mechanism (Toyofuku et al., 2019).

Bacterial extracellular vesicles (BEVs) carry a variety of microbe-associated molecular patterns (MAMPs) and play a key role in host immune recognition. However, no standardized biomarkers can be used to differentiate BEVs specifically from Gram-negative or Gram-positive bacteria. Notably, outer membrane vesicles (OMVs) released by Gram-negative bacteria are often enriched in LPS, in which the lipid A structure can be specifically recognized by Toll-like receptor 4 (TLR4) (Kaparakis-Liaskos and Ferrero, 2015). In contrast, cytoplasmic membrane vesicles of Gram-positive bacterial origin express lipoteichoic acid on the surface of the vesicle, which can activate the TLR2 pathway (Brown et al., 2015; Kaparakis-Liaskos and Ferrero, 2015). In addition, BEVs produced by some pathogenic bacteria are enriched with specific virulence factors. For example, vesicles released by *Porphyromonas gingivalis* contain high levels of LPS, which induces M1-type polarization in host macrophages and enhances pro-inflammatory responses. In contrast, vesicles of *Pseudomonas aeruginosa* origin have been found to induce insulin resistance and impair glucose metabolism functions in skeletal muscle (Choi et al., 2015; Fleetwood et al., 2017). These BEVs play an important role in promoting inflammatory responses and disease processes by delivering virulence molecules to host cells.

**Figure 1:**
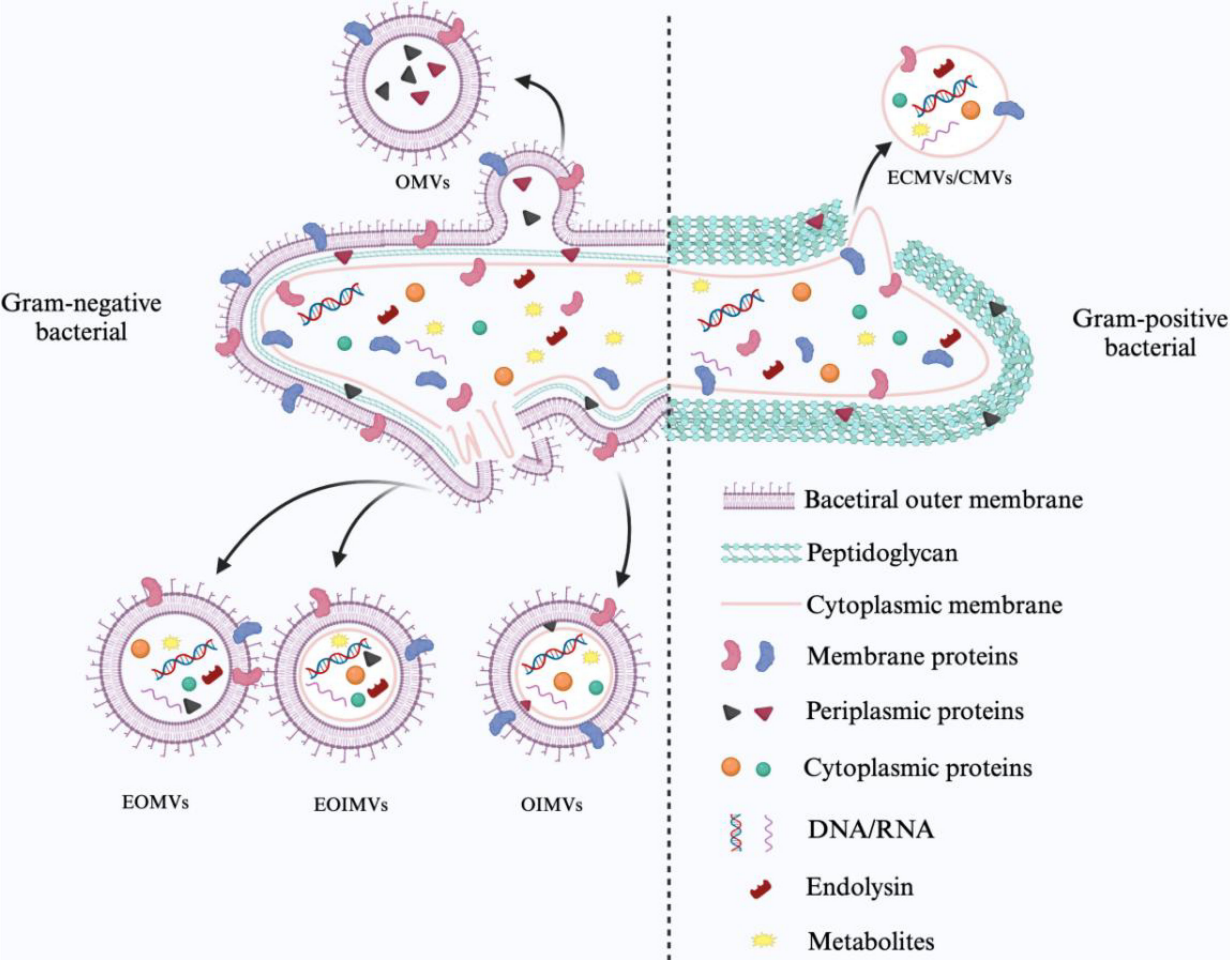
Mechanism of BEVs generation and classification.

It should be noted that the BEVs population itself is highly complex and heterogeneous. Their molecular composition and particle size distribution are not only regulated by the strain’s genetic background and biosynthetic mechanisms but also significantly influenced by a variety of environmental factors, including pH, temperature, nutritional status, and antibiotic exposure, which together regulate the efficiency of vesicle generation, the selective enrichment of bioactive components, and their biological effects on the host (Müller et al., 2021; Ye et al., 2021). Therefore, systematically resolving the dynamic composition of BEVs in different physiological or pathological states is of great significance to promote the standardization of functional studies of BEVs and an in-depth understanding of their mechanisms of action in the host. **Figure 1** briefly summarizes the biogenesis of BEVs of different bacterial origins.

### 2.2 Translocation of BEVs in the gut-liver axis

Studies have shown that BEVs can break through several biological barriers, including the intestinal epithelium, vascular endothelium, and blood-brain barrier, and eventually accumulate in organs such as the liver, lungs, and brain (Jones et al., 2024). For example, *H. pylori*-derived BEVs can co-localize near tight junctions (TJs) and carry the CagA protein, which alters the distribution of closure protein-1 (ZO-1) in the intestinal epithelium and increases the permeability of the intestinal barrier (Turkina et al., 2015). Similarly, BEVs of *Campylobacter jejuni* contain HtrA protease, which cleaves ZO-1 and E-cadherin in intestinal epithelial cells, weakening the integrity of the intestinal barrier (Elmi et al., 2016). These studies have shown that BEVs can increase the permeability of the intestinal barrier, making it easier for bacteria and their metabolites to enter the circulation, affecting distant organs. To more intuitively understand the translocation effect of BEVs, researchers used Cre-recombinase-labeled *E. coli* BEVs and through fluorescent tracer technology, observed that they could be expressed in mouse intestinal epithelial cells (including intestinal stem cells and mucosal immune cells) and fluorescent signals could be detected in organs such as the liver, spleen, kidneys, and the brain, which further confirms that BEVs can be transmitted across tissues in the host body (Jang et al., 2015). In addition, BEVs were similarly observed to enter the blood circulation through dynamin-dependent endothelial cell transport and ultimately accumulate in the liver by oral administration of BEVs to mice (Jones et al., 2020; Schaack et al., 2024).

It is worth noting that the transport of BEVs is not limited to when the intestinal barrier is compromised. Recent studies have shown that BEVs from commensal bacteria are equally capable of crossing the intestinal epithelium into the circulation via the paracellular pathway, even in a healthy state with an intact barrier. BEVs can cross the intestinal barrier relying on kinesin-mediated endocytosis, as well as the paracellular secretory pathway, without the need for disrupting tight junctions (Rubio et al., 2020; Juodeikis et al., 2022; Mottawea et al., 2025). This suggests that BEVs have a selective and controllable ability to cross the barrier. This unique mode of transport provides a new perspective for BEVs to achieve signaling molecules in the presence of a functionally intact intestinal barrier and lays the foundation for their potential role in regulating the intestinal-hepatic axis and remote inter-organ communication.

**Figure 2:**
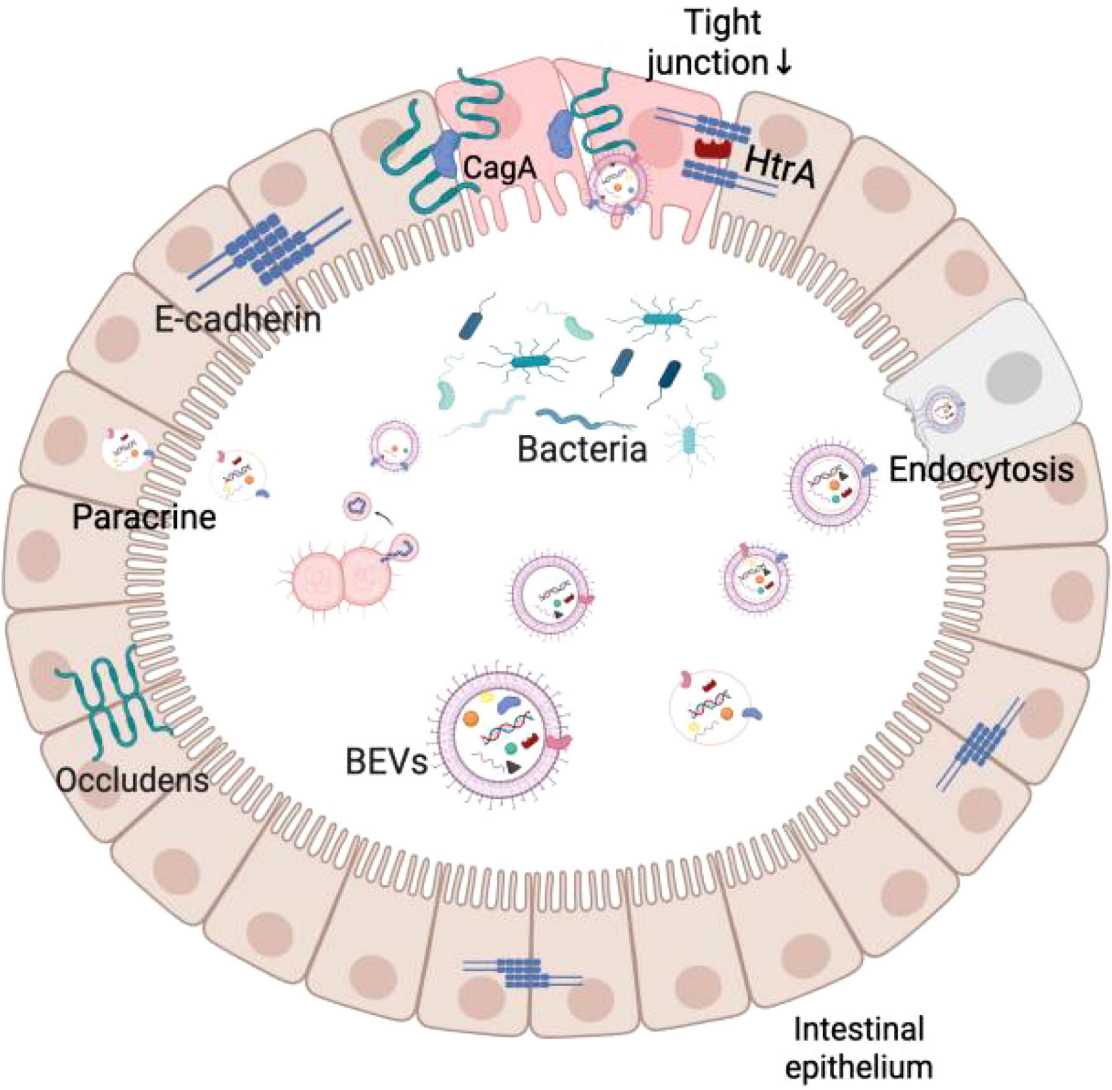
Schematic diagram of BEVs crossing the intestinal mucosal barrier.

When BEVs cross the biological barrier, they interact with host cells through a variety of mechanisms, including direct activation of surface receptors through ligand binding, direct membrane fusion to deliver effector molecules to the cytoplasm or receptor cells through pathways such as giant cytotoxicity, phagocytosis, and endocytosis (Mulcahy et al., 2014; O’Donoghue et al., 2017). Existing studies have shown that BEVs carrying virulence factors can modulate cellular immunity and mediate apoptosis by affecting mitochondrial membrane potential, glucose metabolism, and so on when recognized by host cell receptors (Vanaja et al., 2016; Vitse and Devreese, 2020; Bitto et al., 2021; Balhuizen et al., 2022; Jain et al., 2024). These studies further emphasize that BEVs are not only important signaling mediators for bacteria at the site of colonization but also exert multiple biological effects in distant tissues. **Figure 2** demonstrates a brief overview of BEVs crossing the intestinal barrier.

## 3 The role of BEVs in liver disease

**Figure 3:**
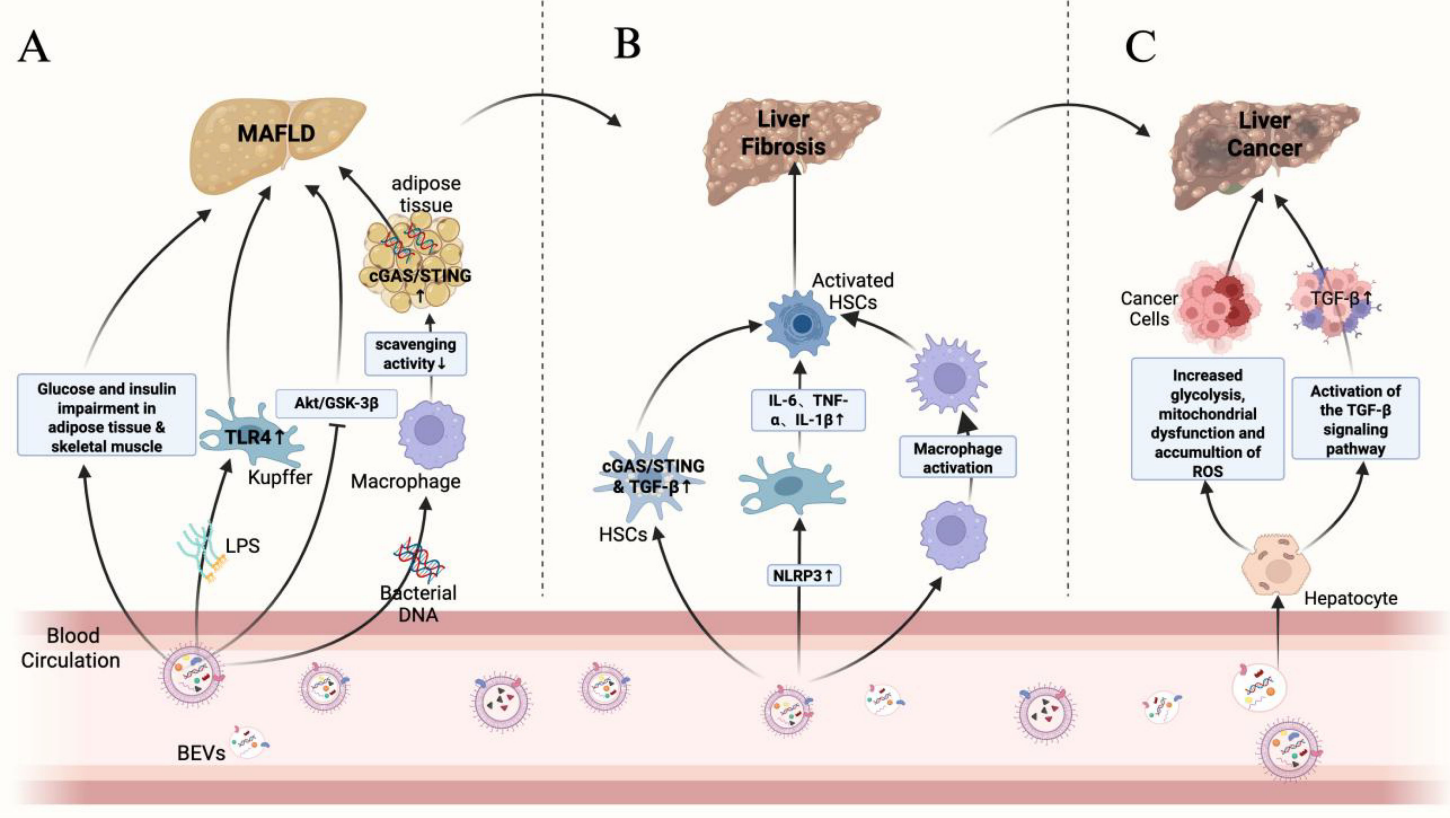
Role of BEVs in different stages of liver disease.

As a complex and dynamically evolving disease, the progression of chronic liver disease usually includes multiple key events, such as hepatocyte injury, release of pro-inflammatory cytokines, immune cell infiltration, activation of hepatic stellate cells (HSCs) with fibrosis formation, which may ultimately lead to cirrhosis and hepatocellular carcinoma (HCC). In this review, we divided the progression of chronic liver disease into three stages: MAFLD/MASH, hepatic fibrosis and cirrhosis, and hepatocellular carcinoma. **Figure 3** demonstrates the role of BEVs in different stages of liver disease. During these stages, BEVs may play multiple roles in the evolution of liver pathology by modulating inflammatory, immune and fibrotic responses, as shown in **Table 1**.

**Table 1:** Role of bacterial extracellular vesicles in various liver diseases.

Type of liver disease	Related nacterial vesicles	Main mechanisms	Key constituent	Reference study
MAFLD	–	cGAS/STING pathway activation; impaired islet B cell function; decreased clearance macrophages	DNA	Luo et al. (2021, 2022)
	*Pseudomonas aeruginosa*	Increased skeletal muscle/adipose tissue insulin resistance; GLUT4 translocation; decreased p-AKT levels	LPS	Choi et al. (2015)
	*Porphyromonas gingivalis*	Inhibition of hepatocyte glycogen synthesis; enhancement of insulin resistance	LPS	Kim et al. (2024); Seyama et al. (2020)
Liver fibrosis	*Helicobacter pylori*	Activation of HSCs; modulation of autophagy in HSCs	CagA、VacA	Zahmatkesh et al. (2022); Bolori et al. (2023); Shegefti et al. (2023)
	*Escherichia coli*	Activation of HSCs; activation of Kupffer cells to release inflammatory factors; induction of macrophage M1-type shift; reduction of albumin synthesis	–	Dorner et al. (2024); Natsui et al. (2023)
Liver cancer	*Helicobacter pylori*	Activation of TGF-β signaling pathway in hepatocellular carcinoma cells	–	Mohammadi Azad et al. (2024)
	*Clostridioides difficile*	Enhances glycolysis; mediates mitochondrial damage; increases intracellular ROS	–	Caballano-Infantes et al. (2023)

### 3.1 The role of BEVs in MAFLD/MASH

Metabolism-associated fatty liver disease (MAFLD), formerly known as nonalcoholic fatty liver disease (NAFLD), occurs mainly in genetically susceptible individuals due to overnutrition and insulin resistance. With the change in lifestyle, the prevalence of obesity and diabetes mellitus continues to rise globally, and MAFLD has become one of the most common chronic liver diseases in the world, with a prevalence of up to 25% (Younossi et al., 2019). Without timely intervention, MAFLD may progress to metabolism-associated steatohepatitis (MASH), liver fibrosis, cirrhosis, and even liver cancer (Younossi et al., 2019).

#### 3.1.1 Role of metabolic disorders due to BEVs in MAFLD/MASH

Gao et al. found that BEVs derived from intestinal flora were enriched in pancreatic islet β cells of obese patients and activated the cGAS/STING signaling pathway via their carried bacterial DNA, thereby promoting pancreatic islet inflammation and impairing insulin secretion by β cells (Gao et al., 2022). In addition, intestinal flora-derived BEVs can deliver bacterial DNA to adipose tissue, exacerbating local inflammation and affecting systemic metabolic functions. Obesity can reduce the number of complement receptors on CRIg^+^ and Vsig4^+^ macrophages in the livers of humans and mice, impairing the body’s ability to clear circulating BEVs. This leads to the accumulation of BEVs, which then spread to adipose tissue and activate metabolic disorders via the cGAS/STING signaling pathway. Furthermore, when BEVs were administered to the intestinal flora of obese mice, insulin-stimulated p-AKT levels significantly decreased, insulin resistance worsened, and the progression of MAFLD was promoted (Luo et al., 2021; Luo et al., 2022).

#### 3.1.2 Role of BEVs-induced inflammatory response in MAFLD/MASH

LPS/TLR4-induced inflammatory response is one of the key factors in MAFLD progression. BEVs from *Pseudomonas aeruginosa* can induce the production of pro-inflammatory cytokines (e.g., TNF-α, IL-6, IL-1β, and MIP-2) and enhance the inflammatory response via TLR4 and partially via TLR2 in mouse macrophages. Animal experiments showed that BEVs from feces induced activation of hepatic stellate cells (HSCs) through the LPS/TLR4 pathway, leading to secretion of inflammatory factors such as IL-6, CXCL1, and CCL2, which promoted hepatic inflammation and accelerated the progression of MAFLD (Ellis et al., 2010; Fizanne et al., 2023). Blocking TLR4 or TLR2 significantly attenuated the inflammatory damage induced by BEVs in feces (Park et al., 2018). Kupffer cells in the liver are hypersensitive to LPS, which activates them to secrete inflammatory factors such as TNF-α and IL-1β. This promotes hepatocellular triglyceride accumulation and indirectly activates HSCs. Activated HSCs accelerate MAFLD progression by secreting pro-fibrotic factors such as TIMP1 and PAI-1 (Miura et al., 2010; Pradere et al., 2010; Nguyen-Lefebvre and Horuzsko, 2015). It was found that after BEVs of *Porphyromonas gingivalis* carrying LPS were injected intraperitoneally, they accumulated in the mouse liver and were mainly taken up by Kupffer cells. Kupffer cells were activated through the TLR4/MyD88 pathway, releasing pro-inflammatory factors such as IL-6, TNF-α, and IL-1β to promote hepatic inflammation, thereby driving the progression of steatohepatopathy. *In vitro* studies have shown that *Porphyromonas gingivalis* BEVs inhibit hepatic glycogen synthesis and enhance insulin resistance, possibly related to inhibition of the AKT/GSK-3β signaling pathway (Furusho et al., 2013; Fleetwood et al., 2017; Seyama et al., 2020; Villard et al., 2021; Kim et al., 2024). In addition, metagenomic analysis of feces from mice fed a high-fat diet showed that BEVs of *Pseudomonas* origin penetrated the intestinal barrier carrying LPS into the liver, skeletal muscle, and adipose tissues. These BEVs inhibited the insulin signaling pathway, reduced insulin-stimulated glucose uptake, lowered the level of p-AKT, and enhanced insulin resistance compared to the regular diet group (Choi et al., 2015). Therefore, we hypothesize that BEVs of at least Gram-negative bacterial origin may be involved in liver injury through activation of the LPS/TLR4 pathway because of the elevated abundance of *Pseudomonas* in the feces of MAFLD patients (Chen and Vitetta, 2020; Solé et al., 2021).

### 3.2 Role of BEVs in liver fibrosis and cirrhosis

If the MAFLD/MASH stage is not effectively managed, patients may go on to develop liver fibrosis. This stage is characterized by the accumulation of collagen in the liver, forming a fibrotic network that limits normal liver function. As hepatic fibrosis progresses, cirrhosis may develop, which manifests as complete structural changes in the liver, including nodule formation and vascular remodeling. Cirrhosis is a critical stage in the loss of liver function and liver failure and a risk factor for hepatocarcinogenesis.

#### 3.2.1 BEVs induce HSCs activation

The ongoing transformation of HSCs from a resting state to a proliferative, fibrogenic and migratory phenotype is a central driver of hepatic fibrosis. Chronic inflammation and metabolic disorders mediated by BEVs are not only the core drivers of MAFLD but also drive the progression of MAFLD to hepatic fibrosis through the activation of HSCs and pro-fibrotic-related signaling pathways. Studies have shown that BEVs of intestinal flora origin can activate the cGAS/STING signaling pathway by carrying flora-originated DNA, which induces the secretion of pro-fibrotic and pro-inflammatory proteins from HSCs and aggravates hepatic fibrosis (Luo et al., 2022; Fizanne et al., 2023).

Existing studies have shown that *Helicobacter pylori(H. pylori)* is a risk factor for the progression of liver disease to cirrhosis (Pellicano et al., 2008). Recent studies have shown that *H. pylori* infection plays a key role in liver disease, especially in non-alcoholic fatty liver disease (Buzás, 2020; Xu et al., 2020). In a study by Zahmatkesh and Bolori et al., it was found that *H. pylori* BEVs containing CagA and VacA factors promoted HSCs activation, whereas BEVs lacking CagA and VacA factors significantly reduced HSCs activation (Bolori et al., 2023; Zahmatkesh et al., 2022). In addition, *H. pylori*-derived BEVs can mediate the progression of hepatic fibrosis by affecting hepatic stellate cell autophagy and regulating hepatic lipid metabolism (Shegefti et al., 2023).

In our previous study, we found that *Escherichia coli* abundance in the feces of cirrhotic patients was significantly higher compared to healthy volunteers (Wang et al., 2023). Dorner et al. showed that when liver-like organs were exposed to BEVs from *E. coli*, the mRNA expression of TGF-β was significantly elevated, and activation of the TGF-β signaling pathway was one of the major driving factors for the activation of HSCs. One of the main drivers of HSCs activation, suggests that *E. coli*-derived BEVs may promote HSCs activation by activating the TGF-β signaling pathway, and further animal experiments revealed that when *E. coli*-derived BEVs were translocated to the liver, the expression of the liver fibrosis markers, α-SMA and Collagen I, was found to be significantly elevated by immunohistochemical staining (Dorner et al., 2024).

#### 3.2.2 BEVs induced phenotypic changes in liver immune cells

An *in vivo* experiment-based study also analyzed the effects of *E. coli*-derived BEVs on hepatocytes and cirrhosis model mice. The results showed that these BEVs induced phenotypic changes in macrophages, promoting the conversion of macrophages to an M1-type pro-inflammatory phenotype through the upregulation of Clec4e expression and indirectly inducing the activation of HSCs through the secretion of pro-inflammatory factors such as IL-1β, IL-6 and TNF-α. They also inhibit the synthesis of albumin in hepatocytes and exacerbate hepatic inflammation. Based on the above experimental findings, researchers further analyzed BEVs in ascites and serum of patients with decompensated cirrhosis, and the results showed that BEVs of intestinal bacterial origin, as well as antibodies against different bacterial antigens, could be detected in the ascites and serum of patients with decompensated cirrhosis (Natsui et al., 2023). In addition, BEVs can indirectly activate HSCs as well as accelerate GSDMD-dependent cell death by upregulating NLRP3-mediated inflammatory vesicle formation, which in turn induces the release of pro-inflammatory cytokines, such as IL-6 and TNF-α, from Kupffer cells and further exacerbates liver fibrosis (Dorner et al., 2024).

### 3.3 Role of BEVs in liver cancer

Hepatocellular carcinoma usually occurs in the setting of cirrhosis. As liver cells continue to be damaged, repair, and regenerate, abnormal cell proliferation may lead to the development of hepatocellular carcinoma (HCC). Hepatocellular carcinoma occurs as the final stage of liver disease and is usually accompanied by dramatic clinical symptoms and a poor prognosis. Therefore, early detection of hepatocellular carcinoma and intervention for early cirrhosis are key to improving patient prognosis.

Bacteria-host interactions play an important role in tumorigenesis and development, and BEVs, as a key mediator between bacteria and host cannot be ignored (Liang et al., 2024). The mechanism of BEVs’ action in tumorigenesis is mainly twofold: firstly, they induce genetic instability in the organism by carrying biologically active substances, such as proteins and nucleic acids; secondly, they stimulate pre-cancerous lesions by activating inflammatory response to stimulate precancerous lesions (Li et al., 2023). Recent studies have shown that the abundance and diversity of BEVs and microbiota in the feces of tumor patients are significantly reduced, but the proteasome carried by BEVs in tumor patients is more diverse and enriched with proteins related to amino acid and carbohydrate metabolism, nucleotide binding, and oxidoreductase activity compared to normal subjects (Mishra et al., 2025). Studies have shown that BEVs are more likely to accumulate in solid tumor tissues such as non-small cell lung cancer, colon adenocarcinoma, melanoma, and breast cancer compared to normal organs (Gujrati et al., 2019; Eyvazi et al., 2020; Kuerban et al., 2020; Basu et al., 2022). These studies reveal the potential role of BEVs in tumorigenesis and development.

Hepatocellular carcinoma, as a solid tumor with a high degree of malignancy, usually involves multiple molecular mechanisms in its development and progression (Yeo et al., 2025). The TGF-β signaling pathway plays an important role in the tumor microenvironment, invasion and metastasis of hepatocellular carcinoma, and blocking the TGF-β signaling pathway can effectively alleviate the development of hepatocellular carcinoma (Xin et al., 2024). In contrast, BEVs can activate the cellular TGF-β signaling pathway through the bioactive substances they carry, activate HSCs, and promote the progression of hepatic fibrosis, and the persistent hepatic fibrous state is a major risk factor for the development of hepatocellular carcinoma (Dhar et al., 2020; Dorner et al., 2024). For example, *H. pylori*-derived BEVs significantly upregulate gene expression in the TGF-β signaling pathway and enhance the invasive ability of HepG2 cells (Mohammadi Azad et al., 2024). In addition, *C. difficile*-derived BEVs can decrease mitochondrial membrane potential and increase intracellular ROS accumulation, which mediates mitochondrial dysfunction, and induce enhanced cellular glycolysis to promote hepatocellular carcinoma progression by up-regulating the expression of the key enzymes of glycolysis, HK2, PDK1, LDHA and PKM2 (Caballano-Infantes et al., 2023).

## 4 The potential value of bacterial extracellular vesicles in the diagnosis and treatment of liver disease

**Figure 4:**
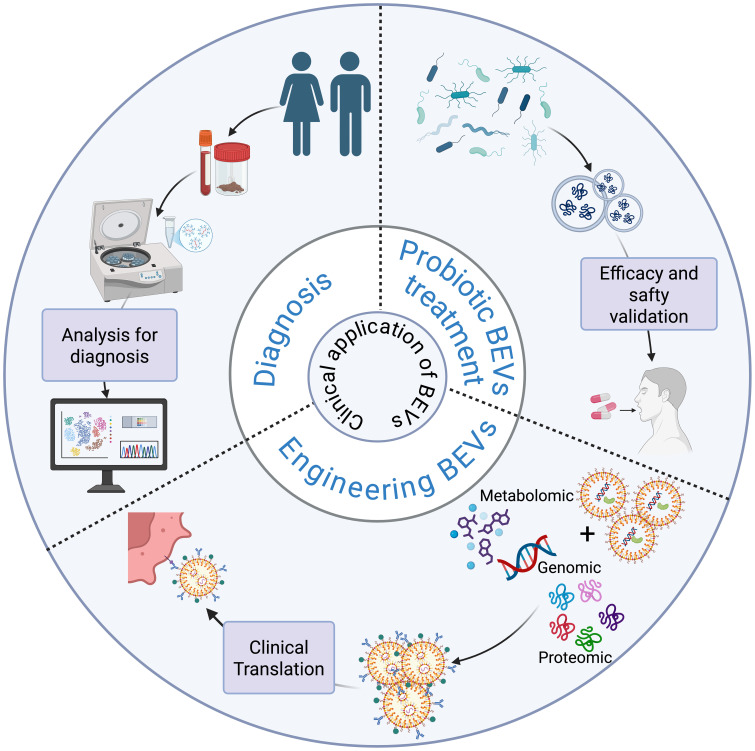
Schematic diagram of the application of BEVs in the diagnosis and treatment of liver diseases.

Extracellular vesicles derived from commensal intestinal bacteria and probiotics have beneficial effects in regulating host physiological functions for the treatment of obesity and insulin resistance. These diseases, often categorized as metabolic syndromes, can serve as etiological and detrimental factors in the development of liver diseases, with many overlapping pathogenic mechanisms and therapeutic approaches. BEVs, as natural immunogenic and non-self-replicating vectors, have a dual role in the development and progression of diseases. On the one hand, BEVs may be involved in the pathogenic process; on the other hand, they can be appropriately modified to achieve diagnosis and treatment of the disease (Kim et al., 2015; Chen et al., 2022). BEVs carry a wide range of parental bacteria as well as bioinformatic molecules and/or metabolic molecules from the survival environment. They are distributed in a variety of body fluids, such as blood, feces, and urine. They can effectively respond to the composition and function of the host bacterial flora through biomolecules involved in bacteria-bacteria and host-bacteria interactions, responding to the pathophysiological status of the host (Yoo et al., 2016; Schaack et al., 2022). Therefore, it is crucial to explore the beneficial role of gut commensal bacteria and probiotic extracellular vesicles in liver diseases. In addition, targeted delivery of drugs and vaccine development utilizing engineered bacterial extracellular vesicles is receiving increasing attention. In this context, we will discuss their ability to diagnose and treat liver diseases. The potential diagnostic role of BEVs in liver diseases is shown in **Figure 4**.

### 4.1 Diagnostic role of extracellular vesicles produced by commensal gut bacteria and probiotics in liver diseases

In the diagnosis of liver diseases (e.g., MAFLD, liver fibrosis, hepatocellular carcinoma, etc.), traditional methods usually require invasive histologic analyses, whereas BEVs, as bacterial-derived extracellular vesicles, not only have the advantages of being easily accessible and non-invasive but also have stronger immunogenicity than host cell-derived vesicles, which provides better specificity in diagnosis (Kaparakis-Liaskos and Ferrero, 2015; Loomba et al., 2021). With the development of multi-omics technologies, BEVs have demonstrated diagnostic potential in the diagnosis of infectious diseases, neurodegenerative diseases, respiratory diseases, and tumors (Dey et al., 2019; Wei et al., 2019; Yang et al., 2020). For example, An Hendrix’s team successfully isolated LPS-positive BEVs from the plasma of patients with intestinal barrier dysfunction and analyzed them quantitatively and qualitatively, and found that the levels of LPS-positive BEVs in the blood of patients with intestinal barrier dysfunction were significantly elevated and positively correlated with the levels of plasma ZO-1 proteins, which implies that the intestinal mucosal integrity of such patients is reduced and permeability is increased, suggesting that they could be used as a potential biomarker of intestinal barrier damage (Jones et al., 2021). In the context of liver disease diagnosis, researchers’ macrogenomic analysis of circulating BEVs from HCC and healthy controls showed an AUC of up to 0.879 based on a model of five genera of BEVs (*Pseudomonas*, *Streptococcus*, *Staphylococcus*, *Bifidobacterium* and *Trabulsiella*) (Cho et al., 2019). Therefore, by effectively isolating BEVs from patients’ body fluids and combining them with multi-omics analysis, it is expected to provide new tools for diagnosis and prognosis prediction of liver diseases in the future.

However, it is worth noting that BEVs from different body fluid sources have their own advantages and disadvantages in terms of their potential for disease diagnosis. Fecal-sourced BEVs can effectively reflect the compositional characteristics of the gut microbiota due to their high yield and diversity, have a strong discriminatory ability in distinguishing disease states from healthy controls, and may even be used to identify different stages or subtypes of diseases, such as the staging of colorectal cancer or the subtyping of inflammatory bowel disease (Park et al., 2021). However, the collection and processing of fecal samples is susceptible to factors such as environmental contamination, and fecal-derived BEVs may be difficult to accurately reflect systemic disease processes or extraintestinal manifestations (Jones et al., 2021; Liu et al., 2024). In contrast, BEVs in peripheral blood have more potential as markers reflecting systemic status, but their biomass is low, the risk of contamination is high, their isolation and characterization still face major technical challenges, and their yield is usually lower than that of fecal samples (Jones et al., 2021; Ou et al., 2023; Liu et al., 2024). Therefore, future studies need to optimize the methods for the detection and analysis of body fluid BEVs further and incorporate longitudinal clinical data with larger sample sizes to comprehensively assess the validity and clinical utility of BEVs as novel disease biomarkers.

### 4.2 Beneficial effects of extracellular vesicles produced by commensal gut bacteria and probiotics

Probiotic therapy plays a key role in liver diseases, and the BEVs secreted by probiotics are inanimate substances that are beneficial to host health, not only avoiding the potential harm caused by live bacteria to the host but also preserving the beneficial and immunogenic properties of the parental bacteria. Therefore, BEVs from probiotics are expected to be novel nanomaterials for the prevention and treatment of corresponding diseases. **Table 2** summarizes the results of studies on the beneficial effects of intestinal symbiosis and probiotic-derived extracellular vesicles on the host, which are mainly related to the regulation of metabolism, the improvement of apoptosis, and the improvement of intestinal barrier function.

**Table 2:** Overview of the impacts of extracellular vesicles derived from commensal and probiotic bacteria on liver diseases.

Biological function	Related bacterial vesicles	Main mechanisms	Reference study
Regulation of metabolism	*Lactobacillus casei*	Regulation of lipid metabolism; regulation of hepatocyte apoptosis Bcl2, Bax expression	Osman et al. (2021); Ye et al. (2023); Behzadi et al. (2017)
	*Akkermansia muciniphila*	Regulates lipid metabolism	Ashrafian et al. (2021)
	*Bifidobacterium (Bifidus)*	Regulates hepatocyte oxidation and inhibits TGF-β signaling pathway activation; attenuates hepatocyte apoptosis and oxidative damage	Li et al. (2024)
Improves inflammation and immunity	*Lactobacillus casei*	Promoted macrophage M2 polarization and myeloid-derived suppressor cell (MDSC) differentiation	Alpdundar Bulut et al. (2020)
	*Akkermansia muciniphila*	Regulation of microRNA expression associated with inflammatory and anti-inflammatory pathways induces production of anti-inflammatory IL-10 in tolerogenic dendritic cells	Mofrad et al. (2024)
Improvement of the intestinal barrier	*Akkermansia muciniphila*	Activation of AMPK, up-regulation of closure protein expression, and reduction of the relative number of harmful bacteria	Chelakkot et al. (2018); Wang et al. (2023)
	*Escherichia coli Nissle 1917*	Regulation of intestinal flora species; elevation of serum SCFA levels; up-regulation of IL-22 transcription and antimicrobial peptide hBD-2	Shi et al. (2023); Alvarez et al. (2016); Fábrega et al. (2016)

#### 4.2.1 Regulation of metabolism

Extracellular membrane vesicles derived from *Lactobacillus* can effectively reduce oxidative damage in hepatocytes, improve lipid metabolism, regulate intestinal flora, and attenuate liver injury, and the therapeutic effect is superior to that of the lipid-modulating drug atorvastatin(ATOR). In addition, *Lactobacillus* and *Bifidobacterium*-sourced BEVs can control tumor progression by inducing apoptosis in hepatocellular carcinoma cells (Behzadi et al., 2017; Osman et al., 2021; Ye et al., 2023; Li et al., 2024). Other researchers have found that oral administration of probiotic strains producing EcEVs resulted in decreased body weight and blood glucose levels in mice, as well as improved metabolism in the intestinal and hepatic systems (Shi et al., 2023). BEVs derived from *Akkermansia muciniphila* can similarly improve obesity by regulating lipid metabolism and reducing weight gain and fat accumulation in HFD mice for the treatment of MAFLD (Ashrafian et al., 2021). However, it is worth noting that the current application of probiotic-derived BEVs in the treatment of liver diseases only remains at the level of *in vitro* cellular and animal experiments, and more clinical studies and technological safety guarantees are still needed in the future.

#### 4.2.2 Modulation of immunity to improve the inflammatory response

Numerous studies have demonstrated that immunomodulatory and anti-inflammatory therapies can effectively alleviate or treat liver diseases, primarily by restoring the balance between pro-inflammatory and anti-inflammatory immune cells within the hepatic immune microenvironment. Immune system dysregulation is recognized as a critical factor contributing to chronic liver disease progression. Hepatic macrophages are broadly classified into the pro-inflammatory M1 and anti-inflammatory M2 phenotypes (Yunna et al., 2020). M1 macrophages can activate hepatic stellate cells (HSCs) and promote collagen synthesis through the TGF-β pathway, while M2 macrophages suppress hepatic inflammation and help slow the development of immune-related liver diseases (Zhang et al., 2022). Additionally, under stimulation by pro-inflammatory and pro-fibrotic cytokines such as TGF-β, IL-6, IL-1β, and TNF-α, macrophages may develop an immunosuppressive, senescence-associated secretory phenotype with pro-fibrogenic characteristics (Matsuda and Seki, 2020). Recent research has shown that exosomes derived from *Lactobacillus plantarum* can drive human THP-1 monocytes toward an anti-inflammatory M2 phenotype, mitigating the inflammatory response of M1 macrophages (Kim et al., 2020). Likewise, bacterial extracellular vesicles from *Lactobacillus casei* have been shown to promote M2 polarization and the differentiation of myeloid-derived suppressor cells (MDSCs), offering anti-inflammatory benefits in various acute inflammation models, including hepatic fibrosis, vaccination, peritonitis, colitis, and wound healing (Alpdundar Bulut et al., 2020). Moreover, Shen et al. found that outer membrane vesicles containing polysaccharide A (PSA) could induce tolerogenic dendritic cells (DCs), enhancing regulatory T cell (Treg) function through PSA-mediated TLR2 signaling via Gadd45α, thereby providing protection in inflammatory diseases (Shen et al., 2012). Similarly, *Akkermansia muciniphila* and its outer membrane vesicles have been shown to promote anti-inflammatory IL-10-producing DCs by regulating microRNAs associated with immune pathways (Mofrad et al., 2024). Collectively, these findings underscore the potential of probiotic and commensal bacterial exosomes to modulate immune cell phenotypes in the liver, highlighting their promise as therapeutic agents for liver diseases.

#### 4.2.3 Improvement of the intestinal barrier

Preserving intestinal barrier integrity is essential for maintaining systemic health. When this barrier is compromised, it has been linked to the onset and progression of liver diseases. Specifically, enhanced intestinal permeability allows microbial components such as LPS and microbial-derived metabolites like bile acids to translocate into the bloodstream, thereby contributing to liver pathology. Barrier disruption is often associated with impaired TJ function (Suzuki, 2013). Chelakkot et al. expanded our understanding of how adherent *Akkermansia muciniphila* can support intestinal barrier integrity. In models using high-fat diet (HFD)-induced dysbiosis in mice and LPS-exposed Caco-2 monolayers, they demonstrated that treatment with exocytosed products from these bacteria activated AMPK signaling, restored occludin levels, and mitigated LPS-induced barrier damage (Chelakkot et al., 2018). Wang et al. reported that extracellular vesicles from *Akkermansia muciniphila* helped rebalance gut microbiota in dysbiotic mice by promoting the growth of beneficial microbes via membrane fusion and reducing harmful species through enhanced mucosal IgA responses. These vesicles also upregulated tight junction protein expression and mucus production, thereby reinforcing the intestinal physical, immune, and biological barriers (Wang et al., 2023). Similarly, Alvarez et al. showed that in human intestinal epithelial cell monolayers, exosomes derived from *Escherichia coli Nissle 1917* and the commensal ECOR63 strain improved barrier function by increasing ZO-1 and claudin-14 levels while decreasing the expression of the pore-forming protein claudin-2 (Alvarez et al., 2016). Moreover, colon tissue analysis revealed that *E. coli strain ECOR12*, when treated with either *E. coli Nissle 1917* or outer membrane vesicles from commensals, led to upregulation of IL-22 and the antimicrobial peptide hBD-2, both essential for reinforcing epithelial defenses (Fábrega et al., 2016). Collectively, these findings suggest that exosomes and outer membrane vesicles derived from probiotics and gut microbes play a key role in enhancing intestinal barrier integrity and may partly explain their protective effects in liver diseases by limiting the translocation of bacterial metabolites into systemic circulation.

### 4.3 Engineered BEV in liver diseases

Much of the current work on the application of engineered BEVs in liver diseases focuses on their role in liver tumors. Disturbances in intestinal flora can alter the immune microenvironment of liver cancer, such as inducing T-cell depletion and immunosuppression, thus increasing tumor susceptibility, and remodeling TME has become an important direction in tumor therapy (Levy et al., 2017; Behary et al., 2021). Some studies have shown that BEVs exhibit great potential in improving the tumor microenvironment. Animal experiments have shown that intravenous injection of genetically engineered modified *E. coli* BEVs can achieve long-term resistance to the immune response of a variety of tumors by relying on interferon-γ, for example, liver tumors, renal tumors, and colorectal tumors, etc., and can completely eradicate established tumors without showing significant side effects. In addition, BEVs are also targeted; when systematically administered, BEVs can be observed to specifically target and accumulate in tumor tissues, subsequently inducing the production of anti-tumor cytokines CXCL10 and interferon-γ in the tumor microenvironment and inducing the aggregation of immune cells, such as NK cells and T cells, so as to achieve the therapeutic purpose (Kim et al., 2017). Surface engineering technology reduces the immunogenicity of BEVs by attaching CD47 to their membranes, remodels the TME by inducing macrophage M1 polarization and blocking immunosuppressive pathways, and the effect also exhibits long-term immune memory in the event of recurrent tumors (Feng et al., 2022). In addition, through drug encapsulation, BEVs are equally effective in inducing phenotypic conversion of macrophages in the TME from M2 to M1, thereby suppressing tumor development (Jiang et al., 2023). Immune checkpoint therapy also plays an important role in treating tumors; however, this approach is often challenged by immune and drug resistance. Studies have shown that BEVs can serve as essential immune adjuvants to overcome this dilemma. By inserting the OM structural domain of PD1 altered on BEVs, they can be made to bind specifically to PD-L1 on tumor cells and protect T cells from PD1/PD-L1 immunosuppression (Li et al., 2020). Currently, more and more studies have shown that BEVs combined with other treatments can further enhance tumor immunotherapy, completely eradicate tumors, and even prevent tumor recurrence and metastasis (Kuerban et al., 2020; Liu et al., 2023). However, it is also important to note that the existing studies still lack clinical trial support, while the natural immunogenicity of BEVs may have an impact on the human body. The rational use of the immunogenicity of BEVs combined with engineered modifications may be a new strategy for tumor therapy.

## 5 Challenges and future prospects

Despite the growing interest in the study of BEVs in liver diseases, their clinical application still faces many challenges. BEVs from intestinal bacteria can cross the intestinal barrier and enter the bloodstream to trigger inflammation or modulate immune responses, but the mechanism of action of BEVs in the liver is not fully understood, and the following challenges still exist in the research and application of BEVs.

### 5.1 Detection of the contents of BEVs

The specific role of bioactive molecules carried by BEVs on host cells has not been clarified. Most of the current studies have focused on the overall effects of BEVs, and fewer studies have delved into which specific class of bioactive substances in BEVs plays a key role in cell damage. Only some studies have also elucidated the damaging effects of LPS and bacterial DNA carried by BEVs on host cells (Tulkens et al., 2020b). Currently, the multi-omics analysis of BEVs only stops at the transcriptome to find the source, and there is no multi-omics joint analysis to elaborate on the specific pathogenic factors of BEVs. Therefore, further study of the downstream components of BEVs through multi-omics, such as genome, proteome and metabolome, is more conducive to the understanding of bacteria-bacteria and bacteria-host interactions and lays the foundation for clarifying their pathogenic mechanisms and regulatory pathways.

### 5.2 Separation of BEVs

How to efficiently separate BEVs from body fluids remains an open question worthy of further exploration. Numerous studies have demonstrated the presence of BEVs in human body fluids, yet no standardized method exists for their separation and detection. Effectively isolating BEVs from various body fluids is a critical step toward their clinical application. Common methods for isolating BEVs include ultracentrifugation, ultrafiltration, size-exclusion chromatography, and density gradient centrifugation (Bauman and Kuehn, 2006; Northrop-Albrecht et al., 2022). Among these, ultracentrifugation is the most commonly used technique, relying on centrifugal speed to separate vesicles from cellular debris, bacteria, and other components. However, the high centrifugal force can damage the structure and function of BEVs, leading to the loss of some bioactive substances. Ultrafiltration utilizes membranes with different molecular weight cutoffs to selectively separate and concentrate vesicles, offering the highest recovery efficiency but low separation purity. Size-exclusion chromatography separates particles of different sizes using a porous polymer gel matrix, achieving high purity but being limited to small sample volumes. Density gradient centrifugation separates BEVs into specific density layers via ultrahigh-speed centrifugation, providing the highest purity but being time-consuming and technically complex, which limits its clinical translation. Tulkens et al. (2020a) proposed an efficient separation method combining density gradient centrifugation with size-exclusion chromatography, which effectively separates bacterial outer membrane vesicles, host cell-derived vesicles, and protein components from human fluids. Although this approach shows clinical feasibility, it also has limitations: density gradient centrifugation may result in the loss of some low-density BEVs, and there is no standardized method for separating BEVs from different body fluids. Therefore, further exploration and optimization of BEVs separation methods—particularly density gradient centrifugation combined with size-exclusion chromatography—are essential for clinical translation. Developing more efficient, standardized, and automated BEVs separation strategies is a core prerequisite for advancing their clinical application.

Following the separation of BEVs, accurate characterization is equally crucial. Commonly used methods include nanoparticle tracking analysis (NTA) for measuring the particle size distribution and concentration of BEVs. However, NTA requires strict detection conditions (e.g., temperature, concentration, calibration) and has limited specificity, as it cannot distinguish BEVs from cellular debris or potential contaminants, let alone differentiate BEVs from various sources. Dynamic light scattering (DLS) can also be used to measure BEV particle size, but it is only suitable for samples with high concentrations and uniform particle sizes. Furthermore, DLS cannot provide information on particle concentration, limiting its specificity (Coelho et al., 2019; Mei et al., 2020). Morphological characterization of BEVs typically relies on transmission electron microscopy (TEM) (Jung and Mun, 2018; Shao et al., 2018). In addition, no universally accepted biomarkers specific to BEVs have been established. Preliminary identification of BEVs from different origins can only be achieved by detecting known lipid compositions, outer membrane proteins, specific genes, or functional proteins (Cao et al., 2020; Díaz-Garrido et al., 2021; Tiku et al., 2021). Therefore, in-depth characterization of BEV composition, as well as identifying their sources and functions, will provide systematic support for BEV research and applications.

### 5.3 Heterogeneity of BEVs

BEVs can be used as biomarkers for the diagnosis and prediction of diseases, and many studies have described their potential as biomarkers for future diseases (Yang et al., 2020; Han et al., 2021; Yoon et al., 2023). However, BEVs are heterogeneous, and BEVs produced from different strain sources as well as different survival environments are usually very different; for example, *H. pylori*-derived BEVs induce hepatic fibrosis progression in liver fibrosis through activation of HSCs as well as modulation of autophagy, but in hepatocellular carcinoma, *H. pylori*-derived BEVs promote tumor progression through activation of the TGF-β signaling pathway (Zahmatkesh et al., 2022; Shegefti et al., 2023; Mohammadi Azad et al., 2024). In addition, while pathogenic *E. coli*-derived BEVs can promote liver fibrosis progression, *E. coli* BEVs derived from the *Nissle 1917* strain can ameliorate liver disease by regulating intestinal flora species and elevating serum SCFA levels (Natsui et al., 2023; Shi et al., 2023). This shows that BEVs of the same bacterial origin have different roles in different diseases, and even BEVs of the same bacterial origin from different strains have different roles in liver diseases. Moreover, there is no effective method to identify specific BEVs from complex body fluids such as feces and blood (Ban et al., 2016; Younossi et al., 2018; Hong et al., 2019). Therefore, more sophisticated technologies such as proteomics, lipidomics or metabolomics are needed to screen for specific “molecular fingerprints” of BEVs for non-invasive early diagnosis (e.g., an alternative to liver puncture), accurate typing (to distinguish viral or alcoholic liver disease) and treatment monitoring.

### 5.4 Storage of BEVs

Maintaining the activity of BEVs requires strict storage conditions, and preserving the activity of isolated BEVs is crucial for advancing subsequent research. Different storage temperatures and durations have varying impacts on BEVs activity (Hong et al., 2019; Noonin et al., 2022). Therefore, optimizing storage and transport conditions tailored to BEVs derived from different body fluid sources is essential for their clinical application. To ensure the functional integrity of BEVs as research materials or therapeutic agents, systematic studies on the optimal storage environment, anti-degradation mechanisms, and transportation methods are needed. Establishing a standardized, high-fidelity, and highly controllable storage protocol will provide a solid foundation for future research and clinical use.

### 5.5 Safety and efficacy of BEVs

The use of live probiotics may increase the risk of intestinal dysbiosis or sepsis, especially in immunocompromised populations (Doron and Snydman, 2015). In contrast, beneficial BEVs offer key advantages: they are non-replicative, can produce effects similar to those of their parental bacteria, and do not carry the risk of live bacteria entering the bloodstream. However, a potential limitation lies in the complexity of probiotic BEV composition. BEVs derived from Gram-negative bacteria retain active components but cannot completely avoid carrying LPS, so safety concerns remain. Therefore, systematic evaluation of bioactive molecules carried by different types of probiotic exosomes is essential to assess potential unintended effects. For example, glycine supplementation has been shown to greatly enhance exosome production from the probiotic *E. coli strain Nissle 1917* while reducing the endotoxin activity of these exosomes (Hirayama and Nakao, 2020). Future BEV production processes should focus on optimizing *in vitro* culture conditions to maximize therapeutic efficacy for specific diseases while minimizing potential side effects. To ensure the effectiveness of BEVs, further studies on their pharmacokinetics and dosage regulation principles are necessary. Given the possible limitations of probiotic BEVs from a single strain in modulating host physiological functions—especially for complex diseases such as liver diseases, which involve multiple pathological processes—the combined application of BEVs from multiple sources may achieve synergistic effects by targeting multiple pathways. This approach may provide a new strategy for the comprehensive treatment of liver diseases, following thorough elucidation of the biological functions and immunomodulatory properties of different probiotic BEVs.

In summary, although the research and application of BEVs still face many challenges, including the lack of efficient isolation and purification techniques, the screening difficulties of specific molecular markers, the insufficient standardization of storage and transportation conditions, and the imperfection of the immunosafety assessment system, their natural immunomodulatory ability, trans-tissue barrier transport ability, and engineered design make them not only expected to become “humoral signal sensors” reflecting the state of liver disease but also likely to be developed into smart therapeutic carriers integrating diagnostic and targeted delivery functions. However, its natural immunomodulatory ability, cross-tissue transport ability and engineered design make it not only a “fluid signal sensor” reflecting the state of liver disease but also a smart therapeutic carrier integrating diagnosis, intervention and targeted delivery. With the deepening of mechanism research and continuous breakthroughs in key technologies, BEVs are expected to play a central role in the precise diagnosis, early intervention and individualized treatment of liver diseases and to drive the entero-liver axis into a new era of “intervention-based microecological medicine.”

## 6 Conclusion

This paper systematically describes the key role of BEVs in the development of liver disease. As an important mediator between digestive tract microorganisms and the host liver, they may be key drivers of disease progression by carrying multiple pathogenic factors across the intestinal barrier and mediating inflammatory responses, metabolic disorders, and fibrotic processes. Notably, BEVs have dual roles: they can act as pathogenic factors to exacerbate liver injury, but also as non-invasive biomarkers and engineered therapeutic vectors, providing new strategies for diagnosis of liver disease and precision intervention. In the future, we can focus on the following directions: (1) analyzing the heterogeneity of BEVs and the functional specificity of the molecules they carry; (2) developing highly efficient isolation techniques and integrated multi-omics analysis methods to screen for molecular markers specific to liver disease; (3) exploring the potential application of engineered BEVs in targeted drug delivery and immune modulation. Through interdisciplinary technological breakthroughs and clinical translational research, BEVs are expected to become an innovative tool in the diagnosis and treatment of liver disease and promote the research of the “gut-liver axis” towards the era of precision medicine.
